# Ibrutinib targets mutant-EGFR kinase with a distinct binding conformation

**DOI:** 10.18632/oncotarget.11951

**Published:** 2016-09-10

**Authors:** Aoli Wang, Xiao-E Yan, Hong Wu, Wenchao Wang, Chen Hu, Cheng Chen, Zheng Zhao, Peng Zhao, Xixiang Li, Li Wang, Beilei Wang, Zi Ye, Jinhua Wang, Chu Wang, Wei Zhang, Nathanael S. Gray, Ellen L. Weisberg, Liang Chen, Jing Liu, Cai-Hong Yun, Qingsong Liu

**Affiliations:** ^1^ High Magnetic Field Laboratory, Chinese Academy of Sciences, Anhui, Hefei 230031, P. R. China; ^2^ University of Science and Technology of China, Anhui, Hefei 230036, P. R. China; ^3^ Institute of Systems Biomedicine, Department of Biophysics, Beijing Key Laboratory of Tumor Systems Biology and Center for Molecular and Translational Medicine, School of Basic Medical Sciences, Peking University Health Science Center, Beijing 100191, P. R. China; ^4^ Hefei Science Center, Chinese Academy of Sciences, Anhui, Hefei 230031, P. R. China; ^5^ Synthetic and Functional Biomolecules Center, Beijing National Laboratory for Molecular Sciences, Key Laboratory of Bioorganic Chemistry and Molecular Engineering of Ministry of Education, College of Chemistry and Molecular Engineering, Peking-Tsinghua Center for Life Sciences, Peking University, Beijing 100871, P. R. China; ^6^ Department of Cancer Biology, Dana-Farber Cancer Institute, Harvard Medical School, Boston, MA 02115, USA; ^7^ Collaborative Innovation Center of Cancer Medicine, National Institute of Biological Sciences, Beijing 102206, P. R. China; ^8^ Department of Medical Oncology, Dana Farber Cancer Institute, Harvard Medical School, Boston, MA 02115, USA; ^9^ Department of Biological Chemistry and Molecular Pharmacology, Harvard Medical School, Boston, MA 02115, USA

**Keywords:** Ibrutinib, EGFR kinase, DFG-in/c-Helix-out, inactive conformation, NSCLC

## Abstract

Ibrutinib, a clinically approved irreversible BTK kinase inhibitor for Mantle Cell Lymphoma (MCL) and Chronic Lymphocytic Leukemia (CLL) etc, has been reported to be potent against EGFR mutant kinase and currently being evaluated in clinic for Non Small Cell Lung Cancer (NSCLC). Through EGFR wt/mutant engineered isogenic BaF3 cell lines we confirmed the irreversible binding mode of Ibrutinib with EGFR wt/mutant kinase via Cys797. However, comparing to typical irreversible EGFR inhibitor, such as WZ4002, the washing-out experiments revealed a much less efficient covalent binding for Ibrutinib. The biochemical binding affinity examination in the EGFR L858R/T790M kinase revealed that, comparing to more efficient irreversible inhibitor WZ4002 (Kd: 0.074 μM), Ibrutinib exhibited less efficient binding (Kd: 0.18 μM). An X-ray crystal structure of EGFR (T790M) in complex with Ibrutinib exhibited a unique DFG-in/c-Helix-out inactive binding conformation, which partially explained the less efficiency of covalent binding and provided insight for further development of highly efficient irreversible binding inhibitor for the EGFR mutant kinase. These results also imply that, unlike the canonical irreversible inhibitor, sustained effective concentration might be required for Ibrutinib in order to achieve the maximal efficacy in the clinic application against EGFR driven NSCLC.

## INTRODUCTION

Ibrutinib, an irreversible BTK kinase inhibitor, has been extensively studied in the B-Cell related malignances due to the effective roles for interruption of the B-Cell Receptor (BCR) mediated signaling pathways [1, 2]. In late 2013 and early 2014 it has been approved for MCL and CLL clinical application respectively [3–6]. Currently it is also being actively evaluated in the clinic for the Diffuse Large B-Cell Lymphoma (DLBCL), Multiple Myeloma (MM) and Acute Myeloid Leukemia (AML) either as a single agent or combination with other chemotherapies or biological therapies [2, 7–9]. Recently Ibrutinib has also been reported to be effective against EGFR mutant Non-Small Cell Lung Cancer (NSCLC) and now being tested in phase I/II clinical trial. [10] Ibrutinib forms a covalent bond with BTK kinase in the Cys481 amino acid to exert the irreversible binding. [11] Proteomic study with ABPP approach also demonstrated that ibrutinib could modify the EGFR wide type kinase via Cys797 in A431 cells [12]. Given the clinical importance of EGFR mutant driven NSCLC, we explored the detailed binding mechanism of Ibrutinib with mutant EGFR (L858R, Del19, L858R/T790M) which revealed a relatively less efficient irreversible binding due to a unique DFG-in/c-Helix-out inactive binding conformation. We envision that insight of this special binding mechanism may help to develop more effective Ibrutinib pharmacophore based EGFR mutant sensitive inhibitors for NSCLC and also direct the proper design of the clinical trial to achieve the maximal efficacy.

## RESULTS

### Ibrutinib irreversibly binds to EGFR mutant via Cys797

Given the fact that both EGFR and BTK kinase bear a similarly accessible cysteine residue (Cys481 in BTK and Cys797 in EGFR) in the hinge binding area, we first tried to determine if ibrutinib binds irreversibly to EGFR mutant as it binds to BTK kinase [13]. We first made a panel of EGFR wt/mutant engineered BaF3 isogenic cell lines whose growth is dependent on either TEL fusion with the EGFR kinase domain (TEL-EGFR) or full-length EGFR (F-EGFR) harboring gain-of-function mutations with or without the Cys797 mutation (C797S). [14] Ibrutinib did not affect wt EGFR-expressing BaF3 cells, wt TEL-EGFR, or the TEL-EGFR (C797S) mutation (GI_50_: > 10 μM). (Table 1) It potently inhibited the TEL-EGFR (L858R) (GI_50_: 0.062 μM) mutation, however significantly lost activity against TEL-EGFR (L858R/C797S) (GI_50_: 3.9 μM). It also exhibited activity against TEL-EGFR (T790M) (GI_50_: 0.16 μM), however lost around 10-fold potency against TEL-EGFR (L858R/T790M) (GI_50_: 1.7 μM). Ibrutinib completely lost activity against cell lines harboring the EGFR C797S mutation. Interestingly, Ibrutinib was approximately 10-fold more potent against full length F-EGFR (L858R) transformed BaF3 cells than the TEL-EGFR (L858R) (GI_50_: 0.004 μM vs. 0.062 μM). Similarly, ibrutinib lost around 10-fold activity against F-EGFR (L858R/T790M) (GI_50_: 0.061 μM) and also significantly lost activity when C797S was introduced (GI_50_: 3.8 μM). A similar trend was observed for the EGFR (Del 19) mutation. These results recapitulated the results observed from the intact cancer cell lines and suggest that ibrutinib is highly potent against EGFR (L858R) and EGFR (Del 19) mutations, however moderately active when the T790M gatekeeper mutation is introduced. Comparably, the reversible version of ibrutinib, *i.e.* PCI-R, lost approximately 20–50 fold activities as compared to ibrutinib itself. (Supplementary Figure S1) Combining the results observed with ABPP approach for EGFR wt in A431 cells, these results suggest that ibrutinib works through formation of a covalent bond with Cys797. Irreversible EGFR inhibitors WZ4002, CO-1686 and AZD9291 demonstrated similar activities, except they also moderately inhibited the growth of wt EGFR-expressing BaF3 cells, indicating potential off-target effects. While reversible EGFR inhibitor exhibited similar trend with PCI-R except that it also potently inhibits the EGFR L858R mutant.

**Table 1:** Ibrutinib anti-proliferation efficacy against EGFR mutant isogenic BaF3 cell lines

Isogenic Cell Line GI50(μ M)	WZ-4002	Gefitinib	Ibrutinib	PCI-R	CO-1686	AZD9291
Structure	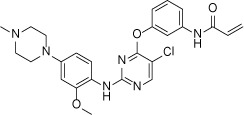	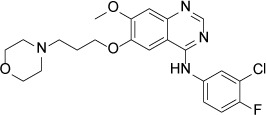	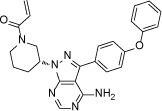	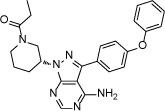	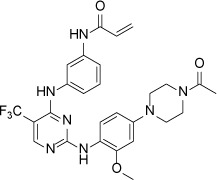	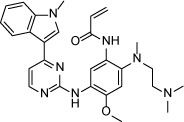
WT-BaF3	> 10	> 10	> 10	7.5	0.97	2.7
BaF3-tel-wt-EGFR	1.6	5.2	> 10	> 10	2.8	1.8
BaF3-tel-WT-EGFR-C797S	1.3	0.11	> 10	> 10	1.3	1.3
BaF3-tel-EGFR-L858R	0.0023	0.0022	0.062	4.7	0.0063	0.0017
BaF3-tel-EGFR-L858R-C797S	3.7	0.006	3.9	9.3	1.6	0.6
BaF3-tel-EGFR-T790M	0.11	> 10	0.16	> 10	0.13	0.022
BaF3-tel-EGFR-T790M-C797S	> 10	> 10	>10	> 10	1.2	1.6
BaF3-tel-EGFR-L858R-T790M	0.0012	2.8	1.7	3.1	0.038	0.007
BaF3-tel-EGFR-T790M-L858R-C797S	4.5	> 10	> 10	> 10	0.88	1.7
BaF3-FL-EGFR-L858R	0.003	0.007	0.004	2.1	0.015	0.002
BaF3-FL-EGFR-L858R-T790M	0.0017	7.2	0.061	1.3	0.0014	0.0021
BaF3-FL-EGFR-T790M-L858R-C797S	1.2	3.7	3.8	3.5	0.73	1.7
BaF3-FL-EGFR-del19- T790M	0.0016	2.9	0.34	1.1	< 0.0003	< 0.0003
BaF3-FL-EGFR-del19-T790M-C797S	0.83	1.6	> 10	> 10	0.63	0.36

### Washing-out experiment revealed ibrutinib's less irreversible inhibition efficiency

**Figure 1:**
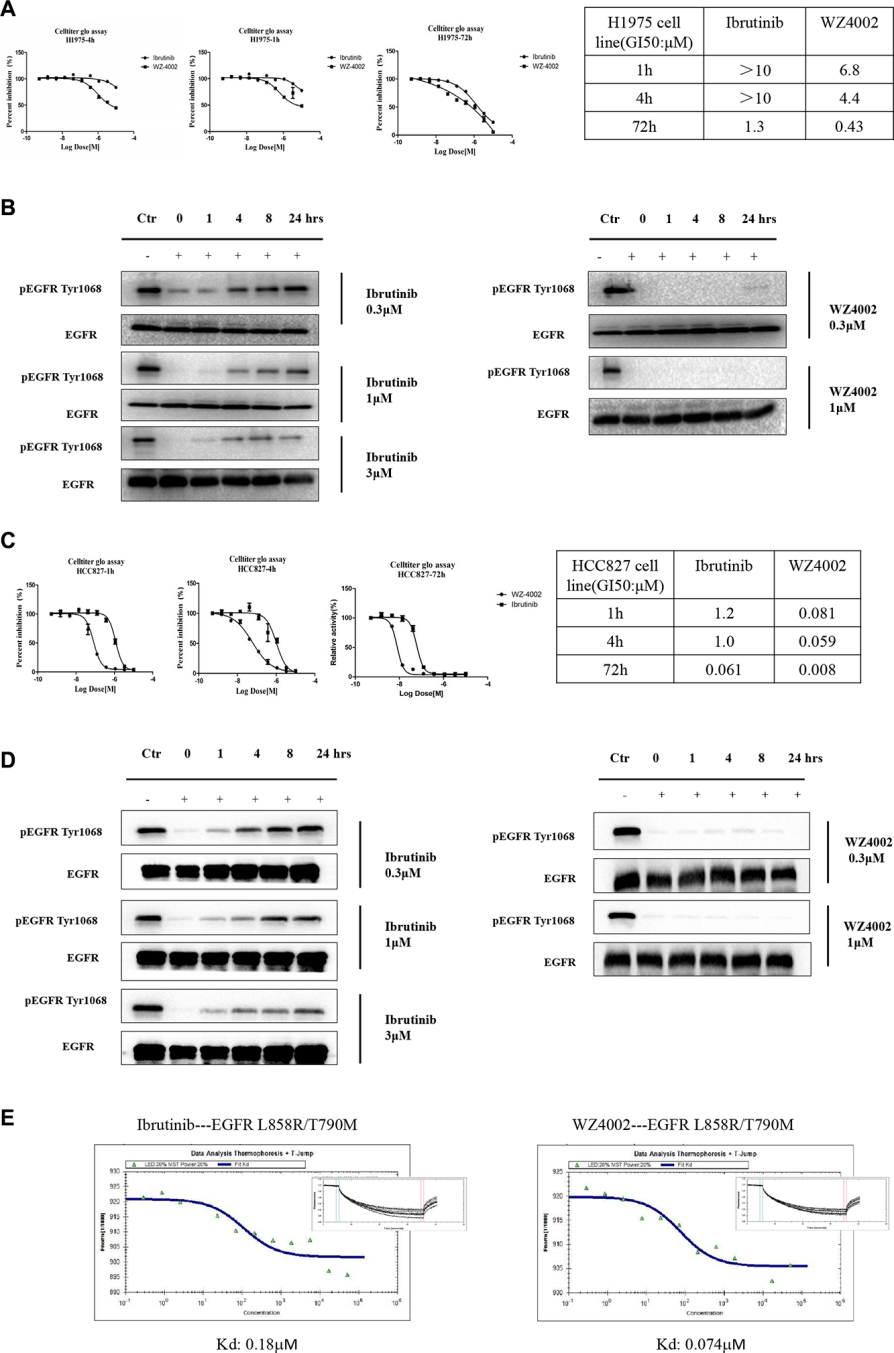
Ibrutinib irreversible binding mode exploration (**A**) Ibrutinib and WZ4002 anti-proliferation effects against the H1975 cell line by removal of drug after 1 h, 4 h and 72 h treatment. (**B**) Ibrutinib and WZ4002 inhibitory effects on EGFRY1068 auto-phosphorylation in the H1975 cell line at different time points by removal of drug after 4 h pretreatment. (**C**) Ibrutinib and WZ4002 anti-proliferation effects against the HCC827 cell line by removal of drug after 1 h, 4 h and 72 h treatment. (**D**) Ibrutinib and WZ4002 inhibitory effects on EGFRY1068 auto-phosphorylation in the HCC827 cell line at different time points by removal of drug after 4 h pretreatment. (**E**) Micro-Scale Thermophoresis (MST) technology based binding Kd test of Ibrutinib and WZ4002 against EGFR T790M/L858R kinase.

To further elucidate the possible irreversible binding mode, we then conducted a washing-out experiment in H1975 cell (EGFR L858R/T790M) using an anti-proliferation assay. The results showed that WZ4002 worked as a typical irreversible inhibitor that exhibited apparent inhibitory activity after even just 1 h pre-drug treatment. However, ibrutinib only exhibited inhibitory activities upon 72 h continuous drug treatment. (Figure 1A) With 4 h pre-drug treatment in the H1975 cell line, WZ4002 (0.3 μM) completely suppressed EGFR Y1068 auto-phosphorylation within 24 h after drug removal. However, at the GI_50_ concentration (1 μM) of ibrutinib, the EGFR Y1068 auto-phosphorylation signal returned following 8 h after drug removal, which suggests a less efficient irreversible inhibition. (Figure 1B) The similar phenotype was observed in the HCC827 cell that harbors EGFR del19 deletion despite of the strong *in vitro* anti-proliferation efficacy comparing to H1975 cells. (Figure 1C, 1D) These results indicated that ibrutinib was a unique irreversible EGFR inhibitor comparing to other typical ones and its inhibitory efficacy might require sustained drug exposure to maintain the signaling pathway suppression. Further testing biochemical binding affinity of ibrutinib with purified EGFR L858R/T790M kinase protein revealed that it beard a binding Kd of 0.18 μM, while more efficient irreversible inhibitor WZ4002 displayed a binding Kd of 0.074 μM. (Figure 1E) This indicated that the less efficiency of the irreversible binding might be due to the less efficient binding.

### Ibrutinib adopted a unique DFG-in/c-Helix-out inactive binding conformation

**Figure 2:**
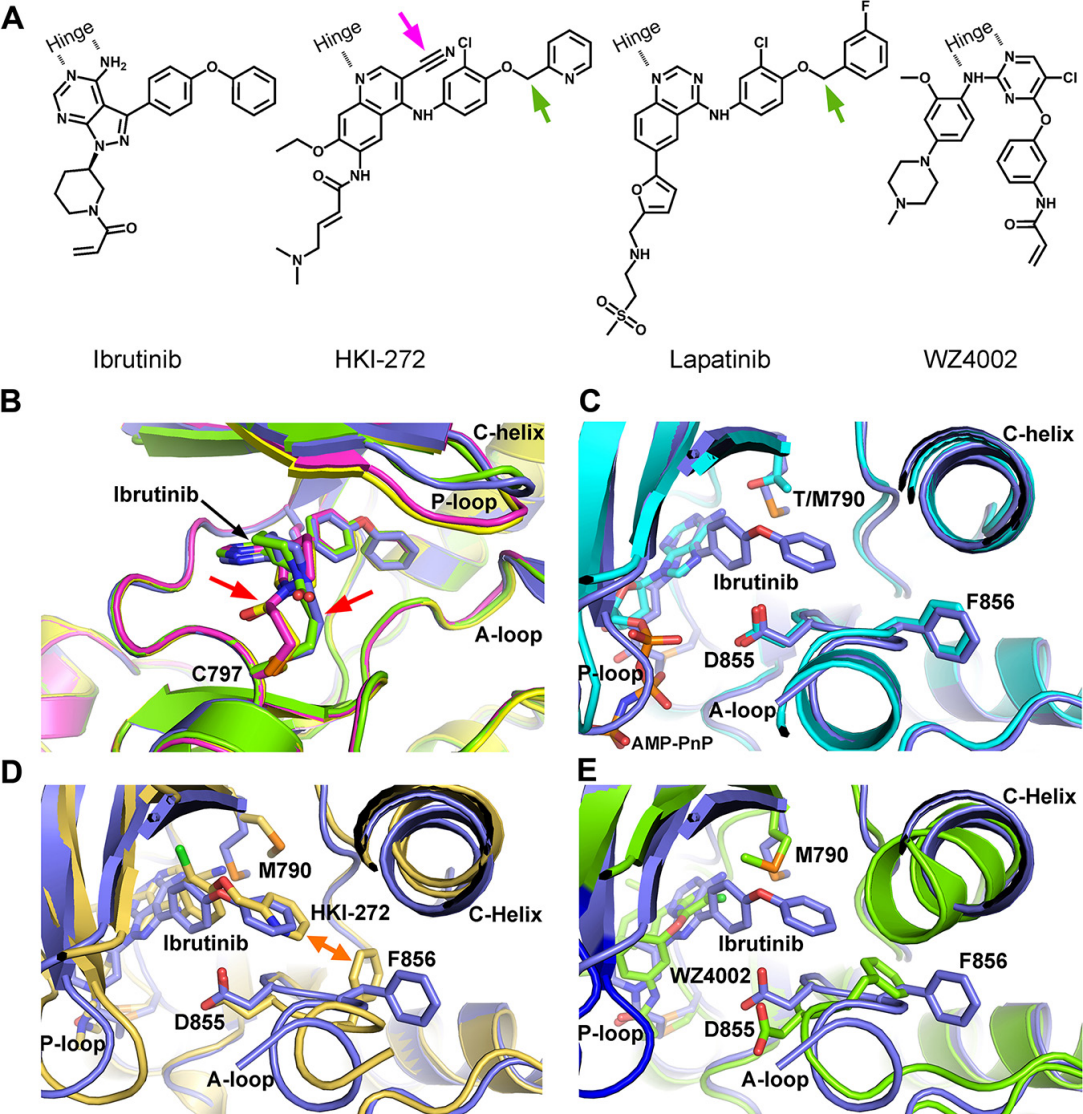
Crystal structure of EGFR T790M+ibrutinib and the comparison with other EGFR T790M+inhibitor co-crystal structures (**A**) Chemical structures of Ibrutinib, HKI-272, Lapatinib and WZ4002 shown in the orientation roughly indicating their binding mode to EGFR. “Hinge” indicates the hinge peptide of EGFR that connects N-lobe and C-lobe of the kinase and interacts with the inhibitors through hydrogen bonds (indicated by dashed lines). The green arrows indicate the extra methylene in HKI-272 and Lapatinib. The purple arrow indicates the cyano group of HKI-272. (**B**) Superimposition of the four molecules in the asymmetric unit of the EGFR T790M+ibrutinib co-crystal structure. The red arrows indicate the slight difference between the two binding modes of ibrutinib. (**C**) Superimposition of the EGFR T790M+ibrutinib structure (slate) and the V948R+AMP-PnP structure (drawn from PDB ID 2GS7, cyan). (**D**) Superimposition of the EGFR T790M+ibrutinib structure (slate) and the T790M+HKI-272 structure (yellow). The double-headed orange arrow indicates the hydrophobic interaction between the inhibitor and the Phe856 side-chain. (**E**) Superimposition of the EGFR T790M+ibrutinib structure (slate) and the T790M+WZ4002 structure (green).

To further explore this special phenotype, we determined a high-resolution crystal structure of EGFR T790M in complex with ibrutinib (PDB ID: 4YNJ, Table 2 and Supplementary Figure S2). Covalent binding of ibrutinib to EGFR Cys797 was confirmed in this structure, and we found that Ibrutinib binds EGFR T790M in inactive conformation, although this protein by itself crystallizes in active conformation [15]. Four EGFR T790M protein molecules were observed in the asymmetric unit of the T790M+Ibrutinib structure, each binding to an ibrutinib molecule. (Figure 2A) Interestingly, despite the same covalent bonds formed between the Cys797 of EGFR and acrylamide of ibrutinib, the four ibrutinib molecules adopt two slightly different conformations in the piperidine-acrylamide moiety. (Figure 2B) The ibrutinib bound EGFR T790M adopts the DFG-in/C-helix-out inactive conformation which closely resembles the previously reported EGFR structure in inactive conformation (PDB ID 2GS7, Figure 2C) [16]. The Met790 side-chain well fits to the inhibitor and make beneficial hydrophobic interaction with the phenyl ring attached to pyrazolopyrimidine. (Figure 2C) This may explain the relative tolerance of ibrutinib to the drug-resistant T790M-bearing EGFR mutants comparing to the first generation inhibitor Gefitinib.

**Table 2:** Data collection and refinement statistics

	T790M + Ibrutinib*
Data collection**	
Space group	C2
Cell dimensions	
*a, b, c* (Å)	168.2, 74.4, 120.5
(°)	90.0, 118.3, 90.0
Resolution (Å)	50.0–1.95 (2.02–1.95)
R_pim_	0.095 (0.450)
*I*/*I*	7.9 (2.3)
Completeness (%)	99.6 (99.8)
Redundancy	3.2 (3.2)
**Refinement**	
Resolution (Å)	41.6–1.95
No. reflections	94949
*R*_work_/*R*_free_	0.200/0.221
No. atoms	
Protein	9690
Ligand/ion	156
Water	950
*B*-factors	
Protein	28.4
Ligand/ion	24.0
Water	37.7
R.m.s. deviations	
Bond lengths (Å)	0.010
Bond angles (°)	1.403
PDB ID	4YNJ

* One crystal was used to collect data for determination of this structure.

** Values in parentheses are for highest-resolution shell.

Since HKI-272 also covalently binds to EGFR in the inactive conformation, we compared the structure of EGFR T790M in complex with ibrutinib to the structure of EGFR T790M in complex with HKI-272 [15]. Two major differences were revealed in the superimposition (Figure 2D) First, the beneficial interaction between Met790 side-chain and the phenyl ring attached to the pyrazolopyrimidine moiety of ibrutinib does not present in the HKI-272 bound EGFR T790M structure. The cyano group attached to the quinolone moiety in HKI-272 prevents this beneficial interaction due to steric hindrance. Second, Ibrutinib does not interact with Phe856 in the DFG motif as HKI-272 and Lapatinib do [15, 17]. One more methylene group presents in the linkage between the terminal pyridine ring and the phenyl ring attached to the quinolone moiety in HKI-272 (similar design applies to Lapatinib). This extra methylene pushes the terminal pyridine (phenyl in the case of Lapatinib) more deeply into the ATP binding pocket, driving it close to the DFG motif. The terminal phenyl thus makes direct hydrophobic interaction with Phe856. This interaction is not observed with ibrutinib since its terminal phenyl ring does not go deeply enough in the ATP binding pocket. This explains why ibrutinib binds EGFR in a distinct DFG-in/C-helix-out conformation, while HKI-272 (and Lapatinib) binds EGFR in a DFG-partially-out/C-helix-out conformation. The relatively weaker binding affinity of ibrutinib to EGFR(L858R/T790M) may be related to this property, too, since the inhibitor-DFG interaction would enhance the binding of the drug.

WZ4002 is another third-generation covalent inhibitor that works well with EGFR T790M. Superimposition of the T790M+Ibrutinib structure with the previously reported T790M+WZ4002 co-crystal structure (PDB ID 3IKA) [18–21] disclosed the distinct difference between these two inhibitors. Ibrutinib forces EGFR kinase to adopt a distinct DFG-in/C-helix-out inactive conformation. While WZ4002 binds to the DFG-partially-out/C-helix-in active conformation. (Figure 2E) The bulky o-bridged biphenyl moiety of ibrutinib can be accommodated by EGFR in the inactive state only, while WZ4002 can bind to EGFR in active state and probably also in inactive state. What's more, to push the EGFR kinase from the ligand binding or mutation (such as L858R) induced active conformation toward inactive conformation would cost extra energy. These may explain why ibrutinib binds to EGFR less efficiently than does WZ4002.

## DISCUSSION

Therefore, we postulated that the transient inhibitory activity of Ibrutinib against mutant EGFR Y1068 phosphorylation might be due to the distinct irreversible binding conformation formation since it required more binding energy to force the protein to adopt the DFG-in/c-Helix-out conformation. This phenotype is concentration dependent. At the same drug concentration, after removal of the drugs from the medium during the washing-out experiment, drug molecules that have already fitted the protein conformation while not yet formed the covalent bond would stay in the protein for longer time (residence time) since the protein required more energy to recover back to the normal state but eventually the drug will be released out from the protein complex due to the equilibrium between the inner cell and cell culture medium. This could explain why during the first several hours the phosphorylation of EGFR was continuously inhibited but then gradually got recovered then. In summary, the less efficiency of sustained inhibition of EGFR phosphorylation was due to the less efficient binding of ibrutinib to EGFR, but the distinct binding conformation gave ibrutinib a longer residence time on EGFR kinase, those ibrutinib molecules which did not form the covalent bond would be gradually released from the protein and hence resulted in the partial of the kinase activity recovery. Therefore an alternative Ibrutinib pharmacophore based scaffold would be needed in order to more efficiently stable the DFG-in/c-Helix out conformation and exert better-sustained inhibitory efficacy for the Y1068 site phosphorylation.

In summary, Ibrutinib inhibits mutant EGFR kinase through formation a covalent bond with Cys797, as other irreversible inhibitors, but due to an unusual DFG-in/c-Helix-out binding conformation, this irreversible binding is much less efficient than other irreversible EGFR inhibitors such as WZ4002. Considering the evidence that ibrutinib is highly potent in *in vitro* for EGFR mutant NSCLC cancer cell lines but only moderately slow down tumor progression in the mouse model, we propose that without alteration of the PK property of Ibrutinib itself, a specially designed formulation or dosage which can help sustain effective concentration should be considered to achieve the efficacy in the clinic application for mutant EGFR driven NSCLC.

## MATERIALS AND METHODS

### Inhibitors

Ibrutinib, W4002, CO-1686, AZD9291, Gefitinib were purchased from Haoyuan Chemexpress Inc. PCI-R was synthesized in the lab based on the procedure reported previously [1].

### Cell lines and cell culture

The human cancer cell lines H1975, HCC827, and A549 cells were purchased from the American Type Culture Collection (ATCC) (Manassas, VA, USA). A431 was purchased from Cobioer Biosciences CO., LTD (Nanjing, China). H1975, HCC827 and EGFR mutant isogenic BaF3 cells lines were cultured in RPMI 1640 media (Corning, USA) with 10% fetal bovine serum (FBS) and supplemented with 2% L-glutamine and 1% penicillin/streptomycin. A549 was cultured in F-12K Nutrient Mixture (kaighn's Modification) (Gibco, USA), and A431 was cultured in DMEM media (Corning, USA) with 10% FBS and supplemented with 2% L-glutamine and 1% pen/strep. H3255 was cultured in BEGM media (LONZA, USA) with 10% FBS and supplemented with 2% L-glutamine and 1% pen/strep. All cell lines were maintained in culture media at 37°C with 5% CO2.

### Antibodies and immunoblotting

The following antibodies were purchased from Cell Signaling Technology (Danvers, MA): EGF Receptor (D38B1) XP® Rabbit mAb(#4267), Phospho-EGF Receptor (Tyr1068) (D7A5) XP® Rabbit mAb (#3777), Antibodies were used at 1:1000.

### BaF3 isogenic cell line generation

Retroviral constructs for Ba/F3-TEL-EGFR and Ba/F3-EGFR variants were made based on the pMSCVpuro (Clontech) backbone. For TEL-fusion vectors, the first 1 kb of human TEL gene with an artificial myristoylation sequence (MGCGCSSHPEDD) was cloned into pMSCVpuro retroviral vector, followed by a 3xFLAG tag sequence and a stop codon. Then the kinase domain coding sequences of EGFR variants were inserted in-frame between TEL and 3xFLAG sequences. For full-length expression vectors, the coding sequences of EGFR variants were directly cloned in pMSCVpuro vector with a 3xFLAG tag at the C-terminal end. All mutations were performed using the QuikChange Site-Directed Mutagenesis Kit (Stratagene) following the manufacturer's instructions. Retrovirus was made using the same method described above and was used to infect BaF3 cells. After puromycin selection, the IL-3 concentration in the medium was gradually withdrawn until cells were able to grow in the absence of IL-3.

### Proliferation studies

Cells were grown in 96-well culture plates (2500–3000/well). For adherent cell lines, compounds of various concentrations were added into the plates after cells were cultured for 12 hours. Cell proliferation was determined after treatment with compounds for 72 hours. Cell viability was measured using the CellTiter–Glo assay (Promega, USA), according to the manufacturer's instructions, and luminescence was measured in a multi-label reader (Envision, PerkinElmer, USA). Data were normalized to control groups (DMSO) and represented by the mean of three independent measurements with standard error < 20%. GI_50_ values were calculated using Prism 5.0 (GraphPad Software, San Diego, CA).

### Washing out experiment

H1975 cells were treated with ibrutinib (0.3 μM, 1 μM, 3 μM), WZ4002 (0.3 μM, 1 μM) for 4 hour before they were thoroughly washed out by PBS for three times. Then cells were incubated in 10% FBS-containing RPMI1640 for indicated time before they are collected and lysed. EGFR, Phospho-EGFR (Tyr1068) antibody (Cell Signaling Technology) were used for immunoblotting.

### EGFR (L858R/T790M) protein purification

A construct encoding EGFR residues 696–1022 with a GST tag was cloned into baculovirus expression vector pAcG2T. The protein was expressed by infecting SF9 cells with high titer viral stocks for 48 h. Cells were harvested and lysed in 25 mM Tris (pH 7.9), 150 mM NaCl, and 1 mM DTT. The supernatant was incubated with glutathione Sepharose beads (Genscript). After washing with wash buffer (40 mM Tris pH 7.9, 500 mM NaCl, 1% Glycerol, 1 mM DTT), the beads were incubated overnight with 5 ml wash buffer containing 5 ul of 5 mg/ ml alpha-thrombin to remove GST tag. The eluted EGFR protein was loaded on desalt column PD-10(GE) to change the buffer to 25 mM Tris pH7.5, 50 mM NaCl, 20 mM MgCl2, and 1 mM DTT. The protein was concentrated to 1 mg/ml and aliquots were frozen and stored at −80°C.

### Biochemical binding Kd examination

The Kd was measured using the Monolith NT.115 from Nanotemper Technologies. Proteins were fluorescently labeled according to the manufacturer's protocol. A range of concentrations of ligands Ibrutinib and WZ4002 (range from 0.05 mM to 2.5 nM) were incubated with 200 nM of purified EGFR T790M/L858R protein 5 min in assay buffer (50 mM Tris, 200 mM NaCl, PH7.4, and 0.05% Tween 20). The sample was loaded into the NanoTemper glass capillaries and microthermophoresis carried out using 20% LED power and 20% MST Power. Kd were calculated using the mass action equation via the NanoTemper software.

### Protein preparation and crystallization

Construct of EGFR kinase domain (residues 696–1022) bearing the T790M mutation was PCR-cloned from the previously made GST-tagged EGFR T790M construct [15] and inserted into pFastBac HTA plasmid between the *Nco*I and *Xho*I restriction sites. Baculoviruse expressing the His-tagged EGFR 696–1022 T790M protein was then generated according to the official protocols of the pFastBac.

The His-tagged EGFR 696-1022 T790M protein was expressed in Sf9 insect cells. After harvesting, the cells were broken by sonication in lysis buffer (20 mM Tris, 150 mM NaCl, 3 mM KCl, 10% glycerol, 1 mM PMSF, 1 mM TCEP, 20 mM Imidazole, pH 8.0, supplemented with Protease Inhibitors Cocktail (Sigma-Aldrich, Cat. S8830). Cell lysate was obtained by centrifugation at 20,000 rpm for 1 hour at 4°C and then applied to the chelating sepharose beads (GE Healthcare, Cat. 17-0575-02) charged with Ni^2+^. The beads were thoroughly washed with wash buffer (20 mM Tris, 500 mM NaCl, 1% glycerol, 0.5 mM TCEP, 30 mM Imidazole, pH 8.0), and then the protein was eluted with elution buffer (20 mM Tris, 500 mM NaCl, 1% glycerol, 0.5 mM TCEP, 300 mM Imidazole, pH 8.0). The affinity tags were removed by cleavage with TEV protease for 3 hours at 4°C, and the untagged EGFR proteins were collected from the flow-through of another pass through the Ni-beads. The eluent was concentrated and applied to gel-filtration chromatography using a Superdex 200 column (GE Healthcare, Cat. 17-5175-01) to further purify the protein. The purified protein was concentrated and dispensed into aliquots, flash-frozen in liquid nitrogen and stored in −80°C freezer for later using.

For co-crystalization, 100 mM ibrutinib solution was added to 8.8 mg/mL EGFR T790M protein solution to achieve the final concentration of 1 mM compound. The mixture was incubated on ice for 2 hours to enable full labeling of EGFR with the compound before setting up the crystallization tray. The crystallization reservoir solution for T790M+ibrutinib was 0.1 M sodium citrate (pH 5.0), 12% PEG 10000, and 3% dioxane, 5 mM TCEP.

### Crystal structure determination and refinement

The T790M+ibrutinib co-crystal diffraction data were collected at beamline BL17U1, Shanghai Synchrotron Radiation Facility at 100K. The X-ray wavelength for data collection was 0.97930Å. The diffraction data were processed with HKL3000 [22]. The structure was solved by molecular replacement method with Phaser [23] using isolated N-lobe and C-lobe of the previously reported EGFR T790M structure (PDB 3IKA) as the search models. Simulated-annealing in CNS [24] was then used to obtain a less biased model and 2Fo-Fc and Fo-Fc maps for manual inspection and adjustment. Repeated rounds of manual refitting and crystallographic refinement were performed using COOT [25] and Phenix [26]. The inhibitor was modeled into the closely fitting positive Fo-Fc electron density and then included in following refinement cycles. Topology and parameter files for the inhibitors were generated using PRODRG [27]. Reported by Ramachandran plots, the percentages of residues in the favored and allowed regions for the final refined structure are 98.1% and 1.9%, respectively. No residues are in the outlier region. The structure was deposited in Protein Data Bank (PDB) with the entry IDs 4YNJ.

## SUPPLEMENTARY MATERIALS


