# Supplementary material for: Ibrutinib targets mutant-EGFR kinase with a distinct binding conformation
Source: Oncotarget. 2016 Sep 10;7(43):69760–9. doi: 10.18632/oncotarget.11951 (PMC5342513; doi:10.18632/oncotarget.11951)
Supplement: Supplementary file 1 [file oncotarget-07-69760-s001.pdf]

Ibrutinib targets mutant-EGFR kinase with a distinct binding conformation

Supplementary Materials

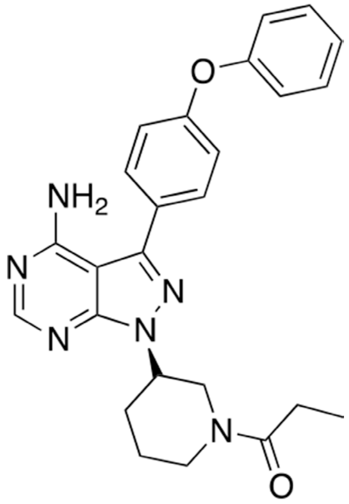

PCI-R

Supplementary Figure S1: Chemical Structure of PCI-R

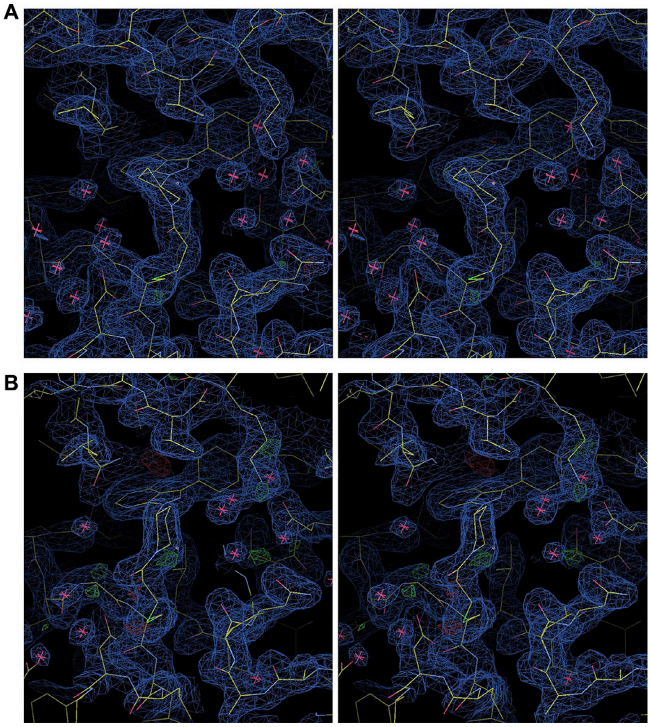

Supplementary Figure S2: Electron density maps of the EGFR 696–1022 T790M+Ibrutinib complex crystal structure. Stereo-view of the electron density (ED) maps are shown. The 2Fo-Fc map (blue) is contoured at 1.2σ, and the Fo-Fc maps are contoured at +3.0σ (green) and −3.0σ (red). (**A**) ED maps around Ibrutinib in Molecule 1 in the asymmetric unit representing the first binding mode of this compound to EGFR. (**B**) ED maps around Ibrutinib in Molecule 3 in the asymmetric unit representing the second binding mode of this compound to EGFR.
